# Good Bread

**Published:** 1869-09

**Authors:** 


### GOOD BREAD.

LIFE ILLUSTRATED says, that the *Boston Bee* says, that WILLIAM HUNT says, in a little book, entitled *Good Bread, or Receipts for Plain Cooking:* "The housewife who has at hand good flour, cold water, and a hot oven, can have on the table, in less than fifteen minutes, enough good, light, delicious bread, for an ordinary family, which may be eaten hot (that is, the bread, not the family), with perfect impunity." If this paragraph had stated where the book was to be had, and at what price it would be sent, post paid, to persons in the country, it might have done some good.

Since the above was written, we made a journey of four miles, riding and walking, in order to obtain a more satisfac-factory report for our subscribers. The book is sent, post paid, by FOWLER & WELLS, New York, for ten cents.

Before giving the recipe for making bread which can be safely eaten while hot, it may be well to premise that hot food, not exceeding a hundred degrees of Fahrenheit, is more healthful than any less warm, because, however cold the food or drink may be which is introduced into the stomach, assimilation, digestion, does not begin to take place until it has been there long enough to acquire a heat of a hundred degrees; and as this heat has to be abstracted from the general system, the whole body suffers, if the person has not a large amount of vitality, which is indicated by a chilly feeling after meals. Some of the most terrible diseases are engendered by eating cold food exclusively for a long time. Food, therefore, which is at least as warm as a hundred degrees of Fahrenheit, is more speedily and more easily digested, and consequently, must be more nutritious and healthful than any eaten colder than that. The sensitiveness of the teeth to cold articles of food, to ice-water, to frozen apples, &c., is a sentinel for safety, as also against anything too hot. Moderately hot bread, therefore, is not injurious, but is positively beneficial. But there is a quality in bread which makes it unwholesome, whether it be hot or cold, and that quality is found in the ordinary hot bread made out of fine flour, and the cold bread made out of corn (Indian) meal—both are sodden; hence, while hot corn-bread, as usually made in the South-west, is healthful, cold corn-bread is certainly unhealthful, in proportion as it is sodden. On the other hand, hot bread, as generally made out of wheaten flour, is unwholesome; not because it is simply hot, but because it is sodden, is heavy, is not light. As, therefore, most persons prefer warm bread, and as it is better, especially in winter-time, that it should be eaten warm, it would seem to be a matter of considerable practical interest to be able to make and bake a batch of wheaten bread within twenty minutes, which can be eaten while hot, not merely with impunity, but with positive advantage. We will therefore give the recipe in full, on the faith of Mr. HUNT's book. We have not tried it, nor do we know that we ever shall; for we are no epicure, we have no nicety of taste as to edibles, and have learned in the exposures of travel, shipwreck, and castaways, to eat almost anything which anybody else has eaten, except onions and tobacco! So our readers must not hold us bound for Mr. HUNT's receipt, nor must they hold him bound, unless they make the bread themselves; and indeed not then, until they have made it quite a number of times, until they have perfected themselves in the art and mystery of making out of good wheaten flour and cold water, with the aid of a hot oven, a healthful light bread, without yeast or powders, and that, too, within fifteen minutes. "The lightness of this bread (called Yankee Rolls), depends very much upon having the oven hot when it begins to bake:

"RECIPE.—Mix good fine flour with pure cold water, and make a dough that can be rolled out and cut into strips, which must again be rolled into a round form, the size of the thumb or finger, and cut into pieces, three or four inches long, bake in a hot or quick oven, ten or fifteen minutes; brown them nicely, and serve fresh.

"This bread being free from yeast, saleratus, soda, or other deleterious substance, will be found, not only light, pure, and sweet, but the most palatable and healthy bread that can be made of fine flour."

This little book, of thirty-one pages, has several dozen receipts for cooking, without physic, and has otherwise valuable information; and we will send a copy of it, free of charge, during September, to every NEW subscriber desiring it, at the time of subscribing.

